# Oxidative Stress Induced by Nuclear Factor Erythroid 2-Related Factor 2 (NRF2) Dysfunction Aggravates Chronic Inflammation Through the NAD^+^/SIRT3 Axis and Promotes Renal Injury in Diabetes

**DOI:** 10.3390/antiox14030267

**Published:** 2025-02-25

**Authors:** Runyuan Li, Xiaoyu Yan, Yuanxin Zhao, Huan Liu, Jian Wang, Yuan Yuan, Qianyuan Li, Jing Su

**Affiliations:** Key Laboratory of Pathobiology, Department of Pathophysiology, Ministry of Education, College of Basical Medical Sciences, Jilin University, 126 Xinmin Street, Changchun 130012, China

**Keywords:** diabetic nephropathy, chronic inflammation, oxidative stress, NRF2, NAD^+^/SIRT3

## Abstract

Diabetic nephropathy (DN), one of the most common and severe microvascular complications of diabetes, significantly increases the risk of renal failure and cardiovascular events. A high-glucose environment can lead to mitochondrial dysfunction in macrophages, which, through remodeling of energy metabolism, mediates the polarization of a pro-inflammatory phenotype and contributes to the formation of a chronic inflammatory microenvironment. Recent studies have found that high-glucose stimulation induces dysregulation of the nuclear factor erythroid 2-related factor 2 (NRF2) redox pathway in macrophages, leading to the generation of oxidative stress (OS) that further drives chronic inflammation. Therefore, it is crucial to fully understand how OS affects macrophage phenotypes and functions following NRF2 inhibition. This review analyzes the role of OS induced by NRF2 dysfunction in the chronic inflammation of DN and explores the relationship between OS and macrophage mitochondrial energy metabolism through the NAD⁺/NADH-SIRT3 axis, providing new therapeutic targets for targeting OS to improve the inflammatory microenvironment and vascular damage in DN.

## 1. Introduction

Type 2 diabetes mellitus (T2DM) has become one of the greatest health crises globally in the 21st century [1]. Diabetic nephropathy (DN), one of the most severe and common microvascular complications of T2DM [2,3], is on the rise in terms of incidence [4]. Approximately 30% to 40% of individuals with diabetes develop DN [5], making it a leading cause of chronic kidney disease and end-stage renal disease [6]. From 1990 to 2021, there was a significant increase in disability-adjusted life years (DALYs) caused by DN, with DN-T2DM increasing by 173.6%. By 2030, the DALYs for DN-T2DM are projected to exceed 14.6 million [7]. Macrophage-mediated chronic inflammation and oxidative stress (OS) have been identified as key triggers for the persistent progression of DN [8,9]. Chronic inflammation, through immune cell infiltration, exacerbates renal damage [10], leading to impairment of the glomerular filtration barrier, mesangial proliferation within the glomeruli, and alterations in the glomerular basement membrane. These changes can even trigger systemic complications such as cardiovascular diseases.

Multiple mitochondrial dysfunctions occur in the kidneys of DN [11,12], which are closely related to chronic inflammation under high-glucose conditions. However, the specific mechanisms between mitochondrial energy metabolism and chronic inflammation remain to be elucidated. Previous studies in our laboratory have shown that macrophage phenotypes are regulated by their metabolic modalities [13,14]. The imbalance in the M1/M2 macrophage phenotype is a key factor contributing to the continued progression of DN, especially the continued activation and pro-inflammatory response of M1 macrophages [15]. In high-glucose environments, macrophages are continuously exposed to pro-inflammatory stimuli and metabolic changes, leading to polarization of the M1 phenotype, which further induces and exacerbates the inflammatory response [15,16]. Specifically, under high-glucose conditions, the activity of nuclear factor kappa B (NF-κB) is enhanced, while nuclear factor erythroid 2-related factor 2 (NRF2) is inhibited and heme oxygenase-1 (HO-1) is downregulated. This leads to increased levels of oxidative stress (OS), with excessive production of reactive oxygen species (ROS) damaging mitochondrial function. Consequently, the balance of NAD⁺/NADH is disrupted, and the silent information regulator 2-related enzyme 3 (SIRT3) is downregulated. These changes drive a metabolic shift in macrophages from oxidative phosphorylation to glycolysis, which is decisive for macrophage phenotypes and plays a key role in the progression of DN.

Currently, an increasing body of evidence supports the critical roles of inflammation and OS in the pathophysiology of DN. In this review, we explore the potential mechanisms by which NRF2 dysfunction leads to OS, which in turn promotes the chronic inflammatory microenvironment in DN through the NAD⁺/NADH-SIRT3 axis, from the perspective of mitochondrial energy metabolism. These findings provide new strategies for the development of targeted therapies and the treatment of DN.

## 2. Activation of Pro-Inflammatory Macrophages in DN Causes a Chronic Inflammatory State That Exacerbates Disease Progression

### 2.1. Recruitment and Stimulation of Macrophage Polarization Towards Pro-Inflammatory Phenotypes in the Presence of High Glucose

Hyperglycemia induces the release of inflammatory mediators after the polarization of macrophages to a pro-inflammatory phenotype, leading to renal injury, which is strongly associated with decreased glomerular filtration rate, histological changes, and poor prognosis [17]. Significantly elevated circulating glucose levels in patients with DN, on the one hand, can activate the NLR family pyrin domain containing 3 (NLRP3) inflammasome to induce pancreatic islet secretion of pro-inflammatory cytokines [18,19], which promotes the recruitment of monocytes and their differentiation into pro-inflammatory macrophages [20], and thus induces inflammatory responses [21]. On the other hand, circulating saturated fatty acids can also be activated by elevated circulating glucose levels, thereby promoting chronic inflammation [22,23,24].

Macrophages are highly plastic as core cells of the immune response, and macrophages are divided into a classically activated M1 state and an alternatively activated M2 state [25]. M1 macrophages promote the inflammatory process by producing pro-inflammatory factors and ROS, whereas M2 macrophages resolve inflammation and induce tissue remodeling by releasing growth factors [26]. M1 and M2 macrophages can switch their phenotypes with each other in specific microenvironments by regulating different transcription factors, such as signal transducers and activators of transcription [27]. Studies have shown that macrophages in streptozotocin-induced DN are predominantly of a pro-inflammatory M1 phenotype [28]. In a study by Ehses et al., it was found that high levels of glucose and free fatty acids stimulated localized proliferation of resident macrophages [29,30,31], which led to a significant increase in the number of CD68^+^ macrophages [32]. In another study, Kamata et al. further found that T2DM increased the accumulation of M1-like macrophages in pancreatic islets [33].

### 2.2. Crosstalk Between Macrophages and Endothelial Cells During DN Leads to Further Amplification of Inflammatory Signals Exacerbating Disease Progression

The progression of DN is closely related to the interaction between macrophages and endothelial cells. It has been shown that in the high-glucose environment of diabetes mellitus (DM), ROS production is increased in endothelial cells and monocytes/macrophages, which may be related to the dysregulation of intracellular NRF2 [34,35]. Increased ROS production activates pro-inflammatory pathways and exacerbates macrophage–endothelial cell interactions [36]. First, ROS in endothelial cells triggers pro-inflammatory activation of endothelial cells by activating *NF-κB* and inhibiting endothelial-type nitric oxide synthase (eNOS). Activated endothelial cells express increased levels of pro-inflammatory cytokines (e.g., TNF-α, MCSF), adhesion molecules (e.g., VCAM-1, ICAM-1), and chemokines (e.g., MCP-1), and the upregulation of these factors promotes macrophage recruitment and migration to the endothelium, further exacerbating and perpetuating the inflammatory response to DM-related vascular dysfunction [37].

**Figure 1:**
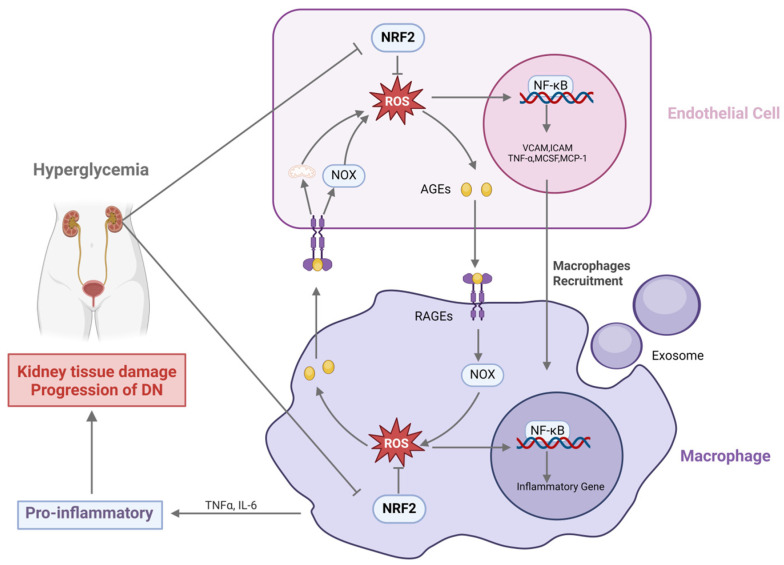
High glucose (HG) increases reactive oxygen species (ROS) production through nuclear factor erythroid 2-related factor 2 (NRF2) reduction, leading to the expression of inflammatory genes such as vascular cell adhesion molecule-1 (*VCAM*), intercellular adhesion molecule (*ICAM*), tumor necrosis factor-alpha (*TNFα*), monocyte chemoattractant protein-1 (*MCP-1*), and macrophage colony-stimulating factor (*MCSF*) by endothelial cells, which induces macrophage recruitment into the lesion. Macrophages secrete exosomes, leading to the downregulation of NRF2 in endothelial cells and increased inflammatory response and oxidative stress (OS). In addition, elevated ROS levels increase the formation of advanced glycosylation end products (AGEs), and AGEs/RAGE interaction further promotes ROS formation via NADPH oxidase (NOX) and mitochondria, leading to activation of ROS-sensitive downstream inflammatory pathways. In macrophages, AGEs/RAGE interaction promotes M1 polarization by inducing secretion of interleukin-6 (IL-6) and TNFα, leading to renal tissue injury and progression of diabetic nephropathy (DN). Created with BioRender.com.

In addition, advanced glycosylation end products (AGEs) play an important role in crosstalk between macrophages and endothelial cells. AGEs bind to their receptor RAGE in an autocrine and paracrine manner, promoting ROS production [38,39]. On the one hand, the expression of the AGE receptor RAGE was enhanced under high-glucose conditions, which activated the NF-κB pathway by activating NADPH oxidase (NOX), as well as the mitochondrial pathway, to increase ROS production, and promoted the upregulation of vascular cell adhesion molecule-1 (VCAM-1) in endothelial cells, which further enhanced endothelial cell recruitment and macrophage migration [40,41]. On the other hand, AGEs secreted by endothelial cells induce oxidative stress and *NF-κB* activation by binding to macrophage RAGE receptors and activating related signaling pathways, which in turn promotes M1 polarization in macrophages [42,43]. Notably, Yiqun Li et al. found that the release of methyltransferase 14 (METTL14) and adipoQ receptor family member 3 (PAQR3) by M1-type macrophages via exosomes was accompanied by the downregulation of NRF2 in human glomerular endothelial cells (GECs), leading to an exacerbation of the inflammatory response and OS in GECs, which exacerbated endothelial cell damage [44]. This macrophage–endothelial cell interaction further promotes macrophage infiltration and M1-type polarization, exacerbating the inflammatory response and ultimately driving the onset and progression of DN (Figure 1).

## 3. Important Role of NRF2 as a Key Protein in DN to Regulate Oxidative Stress and Chronic Inflammation

Inflammation and OS are key mechanisms of diabetic kidney injury [45]. In animal models of DN, increased renal inflammation and OS [46,47] induce glomerular endothelial dysfunction and tethered cell shrinkage, which further leads to glomerular fibrosis and tethered dilatation over time, resulting in decreased renal function [48]. Hyperglycemia induces excessive sorbitol formation via the polyol pathway, AGE, and mitochondrial dysfunction, and leads to excessive ROS production by the mitogen-activated protein kinase (MAPK), poly ADP ribosyl polymerase (PARP), and protein kinase C (PKC) pathways, resulting in the activation of NF-κB, NRF2 [49,50]. Although NRF2 is transiently activated by OS, hyperglycemic stress-induced activation of extracellular-associated kinase (ERK) inhibits the sustained activation of NRF2 [51]. In addition, hyperglycemia leads to a pro-oxidant state by inducing the production of pro-inflammatory factors (e.g., IL-1, IL-6, IL-18, TNF-α, TGF-β, NF-κB, MCP-1, VCAM-1, and ICAM-1), as well as the activation of multiple signaling pathways that, in turn, impair antioxidant defenses. For example, NF-κB activation of macrophage-mediated inflammation enhancement may further impair NRF2 activity and subsequent antioxidant defense [52,53].

In summary, NRF2 plays a crucial antioxidant role in DN, but its activation is limited. Therefore, stimulating the NRF2 pathway and its downstream targets to suppress oxidative stress has become an important therapeutic strategy for DN. Relevant studies have shown that activating the NRF2 signaling pathway and its downstream targets can mediate the inhibition of oxidative stress. For example, Moringa isothiocyanate activates the NRF2/ARE pathway, increasing the gene expression of *GCLC* (glutamate-cysteine ligase catalytic subunit), *NQO1* (NAD(P)H quinone oxidoreductase), and *HO-1*, as well as the protein expression of GCLC and HO-1, thereby reducing ROS levels in high-glucose-treated HK-2 cells in a DN model [54]. Similarly, Notoginsenoside R1 protects db/db mice from DN by upregulating HO-1 expression mediated by NRF2 [55]. These compounds primarily improve OS in DN by enhancing NRF2 activity. Other antioxidants that exhibit anti-oxidative effects under high-glucose conditions may also serve as potential therapies for DN and warrant further investigation (Table 1).

### 3.1. Structure and Function of NRF2

NRF2 is a member of the Cap-n-Collar (CNC) subclass of the basic region leucine zipper (bZIP) cluster and belongs to a family that includes NRF1, NRF3, and NF-E2 p45 subunits [70]. NRF2 is widely found in different species, especially in mammals, and its function is highly conserved. The NRF2 protein consists of seven structural domains (Neh1–7) with different functions [71]. The Neh1 domain contains the bZIP motif, which has been demonstrated to regulate the stability of NRF2, its nuclear translocation, and its interaction with DNA by binding to the antioxidant response element (ARE). The Neh2 domain contains two motifs that interact with kelch-like ECH-associated protein 1 (KEAP1): a strong binding to the ETGE motif and a weak binding to the DLG motif. The Neh2 domain also regulates the proteasomal degradation of NRF2. The Neh6 structural domain is involved in NRF2 degradation independently of KEAP1 through the DSGIS and DSAPGS motifs. The Neh7 structural domain inhibits NRF2 activity by binding to retinoid X receptor-α [72]. The Neh3, Neh4, and Neh5 structural domains have been shown to regulate the transcription of downstream genes [73,74]. NRF2 activity is subject to negative regulation by the cytoplasmic protein KEAP1, which interacts with NRF2 by binding to the KEAP1 and inhibits NRF2 function by binding to the DLG and ETGE motifs of NRF2, whereas specific proteins (e.g., p62 and BRCA2) can relieve this inhibition and promote nuclear translocation of NRF2 [70,75,76,77]. In conclusion, NRF2 plays a central role in cellular antioxidant defense through complex structural domains and regulatory mechanisms.

NRF2-mediated classical antioxidant pathway, Nrf2/Keap1/Are, enhances cellular antioxidant capacity by activating antioxidant genes (e.g., *HO-1*, *NAD(P)H: NQO1*, *GCLC*), scavenging ROS, and attenuating OS [78]. NRF2 also links autophagy to NRF2 signaling through the p62/Keap1/Nrf2 system. p62 activates NRF2 by competitively binding Keap1 and promoting its degradation [79]. Ao Tian et al. found that eicosapentaenoic acid (EPA) ameliorates diabetic complications and cerebral oxidative stress in T2DM mice by increasing p62 levels, degrading Keap1, and promoting NRF2 nuclear translocation [80]. In addition, the NRF2/GPX4 axis plays a key role in antioxidant defense and iron death, and NRF2 inhibits iron death by upregulating glutathione peroxidase 4 (*GPX4*) gene transcription through the ARE [81]. It has been shown that induction of NRF2-mediated GPX4 expression attenuates endothelial cell dysfunction and has anti-DN effects [82]. Interestingly, NRF2-associated epigenetic modifications (e.g., DNA methylation in the Nfe2l2 promoter region) play an important role in oxidative stress regulation, and inhibition of the expression of DNA methyltransferases (DNMTs) reduces Nfe2l2 promoter methylation and enhances its antioxidant function [83,84].

NRF2 not only plays an important role in regulating the antioxidant response but also plays a key role in suppressing the inflammatory response. In an apolipoprotein E-deficient streptozotocin-induced diabetes model, pharmacological activation of NRF2 reduces cytokine production and the accumulation of M1-type macrophages [85]. NRF2 acts as an upstream regulator to directly regulate the transcriptional activities of pro-inflammatory cytokines (e.g., IL-1, IL-6), thereby suppressing the inflammatory response. Kobayashi et al. showed that NRF2 is able to bind to the regulatory regions of inflammatory genes, thereby inhibiting their transcription in macrophages. NRF2 may also indirectly repress transcription of pro-inflammatory cytokine genes by recruiting transcriptional repressors that act transitively on transcription factor complexes formed near inflammatory cytokine genes [86]. In addition, the NRF2 pathway inhibits the activation of the NF-κB pathway by increasing antioxidant defense and target gene *HO-1* expression, thereby reducing ROS-mediated NF-κB activation [87]. Under high-glucose conditions, NRF2 is a key regulator of both anti-inflammatory and antioxidant pathways, which are crucial for cellular protection. The upregulation of NRF2-dependent antioxidants can alleviate systemic oxidative overload and renal inflammation.

### 3.2. NRF2 Inhibition in the Presence of High-Glucose Leads to OS

Several studies have shown that NRF2 expression levels and biological activity are significantly suppressed in high-glucose states [88,89]. Early in DM, NRF2 adaptively attempts to maintain function in order to overcome the diabetic damage; however, in late diabetes, the antioxidant function is further impaired, leading to reduced NRF2 expression [90]. Increases in the nuclear regulators glycogen synthase kinase-3 beta (GSK3β) and Bach1 have been associated with limiting NRF2 activation [91,92], and nuclear GSK3β is increased in advanced T2DM, potentially inhibiting NRF2 activation [52]. In 6-month diabetic hearts, the potential of nuclear NRF2 to bind to EpRE and initiate target gene transcription during advanced diabetes may be inhibited by Bach1 competition. In addition, Weiying Guo et al. demonstrated that mouse double minute 2 (MDM2) prevented P53 inhibition of NRF2 expression and function-induced podocyte loss, as well as thylakoid cell injury. However, MDM2 expression was reduced and P53 was elevated in the renal tissues of DN patients, which was responsible for DM-induced glomerular hypertrophy and fibrosis accumulation [93].

Interestingly, impairment of NRF2 in T2DM is associated with activation of NF-κB [52]. Yi Tan et al. demonstrated that 3-nitrotyrosine accumulation and ERK phosphorylation were significantly increased in the hearts of streptozotocin-induced diabetic mice and diabetic patients with a significant decrease in NRF2 expression and that ERK acts as a negative regulator of glucose uptake by mediating the inhibition of NRF2 activity leading to OS-induced insulin resistance [51]. Furthermore, Qian Zhou et al. showed that the natural flavonoid apigenin (API) upregulated antioxidant defense molecules in AGEs-induced Huvec by inhibiting the ERK/NF-κB signaling pathway triggered by AGEs-RAGE interactions and inducing ERK/NRF2 pathway [94].

The NRF2 redox pathway is dysregulated in macrophages in the diabetic microenvironment. It has been shown that elevated glucose levels and pro-inflammatory cytokine expression in macrophages in vitro inhibit downstream target gene and protein expression (e.g., *HO-1*), significantly reduce NRF2 activation, and upstream phosphorylation of NRF2 by Akt and CD206 is also reduced, and that defects in NRF2 signaling in macrophages in the diabetic environment are more severe than those induced by cytokines alone [35]. Specifically, decreased NRF2 activity in DN from various causes leads to increased neuroinflammation and nitrosative-OS [52]. In addition to DN, NRF2 is also suppressed in chronic obstructive pulmonary disease, one of the typical chronic inflammatory diseases, which may be related to persistent oxidative stimuli leading to depletion of the antioxidant response and reduction in its master regulator, NRF2, and related downstream signaling [95]. These findings emphasize the important role of OS damage resulting from NRF2 inhibition in chronic inflammatory diseases. In the following, we will discuss how OS contributes to increased inflammation and disease progression by affecting macrophage mitochondrial function and energy metabolism, which in turn leads to increased inflammation.

### 3.3. OS Affects NAD^+^/NADH Homeostasis to Downregulate SIRT3 Activity to Mediate Metabolic Reprogramming in Macrophages

OS is a key component of DN pathogenesis, and mitochondrial dysfunction in DN patients is closely related to ROS accumulation due to OS. Increased ROS production has been demonstrated in vitro and in vivo in a variety of mouse models of DN [12], and ROS leads to marked dysfunction of mitochondrial respiratory chain complex I (NADH-ubiquinone oxidoreductase), as well as a decrease in the activity of complexes II, IV, and V (ATP synthases), accompanied by a decrease in the NAD^+^/NADH ratio [96,97], as the NAD-dependent deacetylase Sirtuin3 (SIRT3) plays a key role in deacetylating and modifying the enzymatic activity of respiratory chain complexes I, II, and III (ubiquinol–cytochrome c oxidoreductase). It has been shown that SIRT3 protein levels are significantly reduced in tissues of mice with type 2 DM [98]. Loss of SIRT3 increases ROS production and hyperacetylation of mitochondrial respiratory chain complexes I and III [99,100], accompanied by enhanced glycolytic metabolism [101]. In summary, in DN, OS leads to mitochondrial dysfunction in macrophages, which disrupts the NAD⁺/NADH balance and downregulates SIRT3, thereby mediating metabolic reprogramming.

#### 3.3.1. Role of SIRT3 in Macrophage Mitochondrial Energy Metabolism

A growing body of evidence supports the role of sirtuins in the regulation of cellular homeostasis, particularly metabolism and chronic inflammatory states [102,103]. SIRT3 is a key point in the balance between glycolysis and mitochondrial oxidative metabolism [104], and it regulates mitochondrial function through a variety of mechanisms including direct binding to electron transport chain (ETC) proteins, regulation of mitochondrial dynamics, maintenance of redox balance, and regulating the tricarboxylic acid (TCA) cycle. Shuhui Dai et al. found that in a mouse model of cerebral hemorrhage, intermittent fasting (IF) suppressed microglia activation-induced inflammation by up-regulating the expression of SIRT3 through the SIRT3/NRF2/HO-1 pathway and that SIRT3 may be important in the metabolic change to oxidative phosphorylation and the maintenance of stable mitochondrial function as a result of IF [105].

SIRT3 also regulates energy production by modulating the activities of various enzymes important in the mitochondrial energy metabolism pathway. SIRT3 acts as a ROS inhibitor mainly through the deacetylation of major mitochondrial antioxidant enzymes, such as isocitrate dehydrogenase 2 (IDH2), superoxide dismutase, and glutathione peroxidase [106,107]. SIRT3 has been shown to inhibit NLRP3 inflammatory vesicle activation by activating superoxide dismutase-2 (SOD2) to alter macrophage metabolic reprogramming, which in turn regulates the inflammatory response [108,109]. In conclusion, SIRT3 plays a crucial role in maintaining macrophage mitochondrial bioenergetics, reducing ROS formation, and modulating pro-inflammatory responses.

#### 3.3.2. Effects of OS-Induced ROS Accumulation in DN on NAD^+^ and Its Downstream Key Protein SIRT3

NAD^+^ is not only a core molecule in cellular energy metabolism, but also a key factor influencing mitochondrial function and maintaining physiological homeostasis. Recent studies have shown that NAD^+^ metabolism plays an important role in regulating immune function and inflammation. Yao Zhao et al. constructed an in vivo and in vitro model of chronic cerebral hypoperfusion, and they found that ROS levels were elevated in the modeled group, whereas NAD^+^ administration reduced pro-inflammatory cytokine and ROS production in BV2 microglia through activation of the Sirt1/PGC-1α pathway, which was protective against hypoxia-induced neuroinflammation and mitochondrial damage [110]. A related study showed similar results, indicating that increasing NAD^+^ levels attenuated mitochondrial dysfunction and pro-inflammatory activation in peripheral blood mononuclear cells in heart failure [111].

Disordered NRF2 in DN leads to the accumulation of OS and ROS. Impairment of mitochondrial function causes redox imbalance of NAD^+^/NADH [112,113,114,115,116]. Impaired mitochondrial complexes due to the overproduction of oxidants at DN cause, etc., dysfunction [117]. ROS can react with unsaturated fatty acids in mitochondrial membrane lipids and inhibit the activity of electron transport chain complexes. Peroxidation of cardiolipin has been shown to cause dysfunction of complex I in the diabetic state, as well as a decrease in the stability of supercomplex assemblies in mitochondrial membranes [118]. Excessive ROS production induces oxidative damage to mtDNA, which in turn leads to defective respiratory chain complexes and complete mitochondrial dysfunction [119]. Mitochondrial complex I plays an important role in cellular NAD^+^/NADH homeostasis [120]. The major sources of ROS in mitochondria are the electron transport chain complexes I and III. In these complexes, molecular oxygen is reduced to superoxide anion radicals (O^2−^). Superoxide is also formed during reverse electron transfer from ubiquinol to complex I in over-reduced ETCs, resulting in the reduction in NAD^+^ to NADH [121].

SIRT3 is a key regulatory protein that senses NAD^+^ levels (NAD^+^/NADH ratio) [122]. Reduced NAD^+^/NADH ratio due to complex I deficiency inhibits SIRT3 activity, leading to increased protein acetylation and sensitization of the mitochondrial permeability transition (mPTP) [106]. Investigators have shown that adding exogenous NAD^+^ to glomerular mesangial cells incubated with high glucose maintained the intracellular NAD^+^/NADH ratio and activated the SIRT3-AMPK-mTOR pathway, which blocks glomerular mesangial hypertrophy [123]. Another experiment showed that in complex I-deficient conditional knockout mice, the NAD^+^/NADH ratio and SIRT3 activity were reduced, leading to increased protein acetylation, which accelerated the progression of heart failure [124].

**Figure 2:**
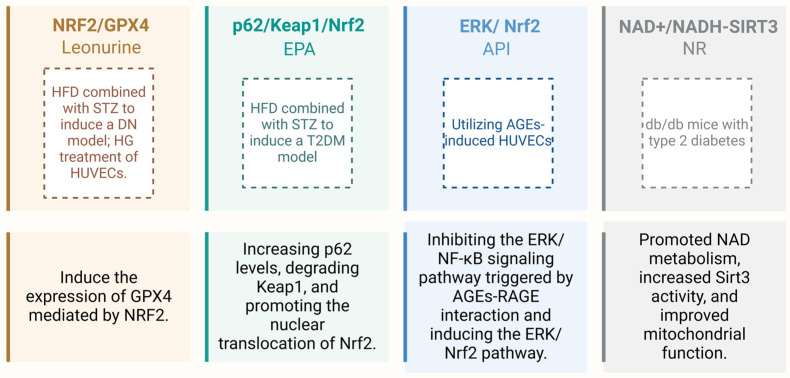
This review cites examples related to NRF2 and the NAD⁺/NADH-SIRT3 axis under high-glucose conditions. Created with BioRender.com.

OS is negatively correlated with NAD^+^ in DN, with NAD^+^ depletion in the matrix [125], and SIRT3 downregulation [126]. It has been reported that SIRT3 expression is significantly reduced in the kidneys of BTBR ob/ob mice with type 2 diabetes mellitus, which correlates with its reduced activity and elevated ROS levels. Administration of SIRT3 agonists showed that the protective effect of SIRT3 on glomeruli was mediated in part by increased SIRT3 tubular expression and upregulation of tubular nicotinamide phosphoribosyltransferase (Nampt) [127]. Komuraiah Myakala et al. found that treatment of type 2 diabetes in db/db mice with nicotinamide riboside (NR) promoted NAD metabolism, increased SIRT3 activity improved mitochondrial function, and reduced inflammation, thereby preventing the progression of DN [45] (Figure 2). In addition, caloric restriction, which can increase healthy lifespan and interventions to prevent metabolic syndrome, reduces OS, leads to increased NAD levels, and improves mitochondrial function through SIRT3-mediated increases in SOD2 activity [117].

In conclusion, NRF2 inhibition leads to OS production and ROS accumulation to disrupt mitochondrial function NAD^+^/NADH balance, and NAD^+^ levels directly control SIRT3 activity. Therefore, it is reasonable to speculate that NRF2 inhibition in macrophages under high-glucose conditions leads to mitochondrial dysfunction and decreases the NAD^+^/NADH ratio to downregulate SIRT3.

However, the indirect linkage between NRF2 and the NAD⁺/NADH-SIRT3 axis via OS is rather complex, as the NAD⁺/NADH-SIRT3 axis is influenced by a variety of metabolic factors. Therefore, further research is needed to elucidate this potential pathway in DN, including factors upstream of NRF2 or regulators between NRF2 and the NAD⁺/NADH-SIRT3 axis. Additionally, in terms of clinical application, the antioxidant regulation of NRF2 is relatively broad, and changes in immune cells may affect intercellular communication. Thus, further in-depth studies are required for the clinical translation of intervention strategies targeting NRF2.

### 3.4. Altered Energy Metabolism Remodels the Pro-Inflammatory Phenotype of Macrophages Driving Dysregulation of the Inflammatory Microenvironment Leading to Sustained Progression of DN

Mitochondria play an important role in macrophage remodeling as a central hub regulating innate immune signaling pathways. Changes in mitochondrial energy metabolism are closely related to macrophage polarization phenotype [15], and damaged mitochondria will induce signals that activate inflammasomes. M1 macrophage metabolism is characterized by enhanced glycolysis, PPP flux, fatty acid synthesis, and truncation of the TCA cycle, leading to the accumulation of succinate and citrate [128]. With prolonged exposure to changes in energy metabolism and pro-inflammatory stimuli, macrophages are reprogrammed to promote an inflammatory response with the release of pro-inflammatory cytokines and chemokines [129], leading to the development of chronic inflammation [15,16]. Thus, manipulation of mitochondrial homeostasis is critical for regulating the metabolic response of macrophages as well as the inflammatory state.

Reduced activity of SIRT3 in DN leads to decreased fatty acid oxidation, disturbed energy metabolism, impaired mitochondrial respiration and oxidative phosphorylation, and polarization of chemotactic macrophages toward a pro-inflammatory phenotype [97]. Research indicates that Sirt3 influences the progression of inflammatory organ damage by modulating mitochondrial function and immune cell activity [108,130]. Deficiencies in Sirt3 lead to mitochondrial dysfunction and excessive activation of the NLRP3 inflammasome, which, in turn, drives macrophages to shift their metabolism from oxidative phosphorylation to glycolysis. This metabolic reprogramming increases the production of pro-inflammatory cytokines, thereby exacerbating inflammatory responses and tissue damage.

**Figure 3:**
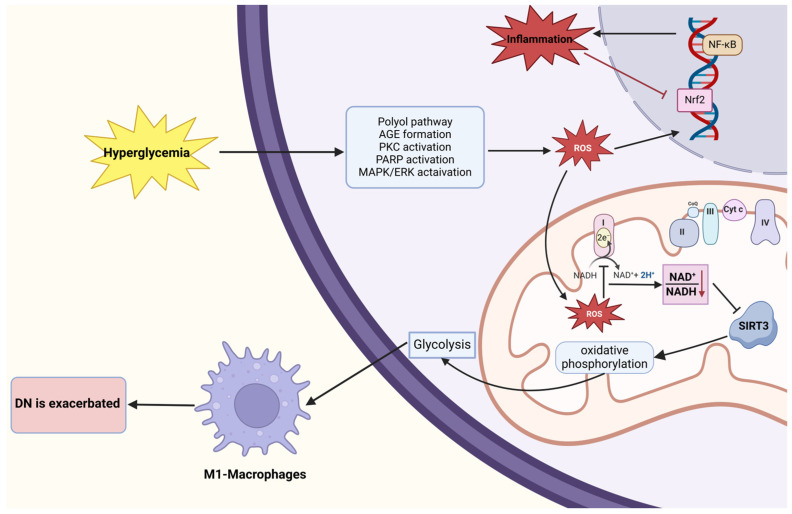
HG leads to inhibition of NRF2, increased ROS production, and decreased NAD⁺/NADH ratio, which in turn downregulates SIRT3 expression and activity, contributing to a shift in mitochondrial metabolism from oxidative phosphorylation to glycolysis and reprogramming to drive sustained activation of the pro-inflammatory macrophage phenotype, type M1, leading to exacerbation of DN. Created with BioRender.com.

In the chronic inflammatory conditions of DN, M1 and M2 macrophages coexist [131,132], and regulate the inflammatory response through polarization status and secreted cytokines, so the imbalance of M1/M2 macrophage phenotype may be a key point in DN. In a recent study of single-cell RNA sequencing, it was shown that changes in the M1 and M2 profiles in macrophage subpopulations within or around pancreatic islets are not highly heterogeneous and that the nature of inflammation during the development of DN is likely to be determined by a mixture of M1- and M2-like macrophages, which largely depends on the balance between activating and inhibitory signals and the tissue environment [26]. In summary, altered energy metabolism remodels the macrophage phenotype, leading to a sustained disruption of the M1/M2 balance and dysregulation of the inflammatory microenvironment, which in turn promotes the development of diabetic kidney injury and multiple complications [133] (Figure 3).

## 4. Conclusions

In conclusion, as one of the microvascular complications of diabetes mellitus, OS is induced by the inhibition of NRF2 activation during DN for various reasons. The accumulation of ROS and RNS in the DN microenvironment leads to the impairment of the mitochondrial electron respiratory chain, as well as the disruption of the NAD^+^/NADH balance, which downregulates SIRT3 expression and activity, affects the reprogramming of mitochondrial energy metabolism, and drives the sustained activation of the pro-inflammatory macrophage phenotype type M1, which mediates the persistent generation of chronic inflammation [13], thus exacerbating the progression of DN.

The antioxidant defense system in the human body is categorized into enzymatic and non-enzymatic pathways. Enzymatic antioxidants, such as SOD and GPX, serve as the first line of defense against OS, while non-enzymatic antioxidants act as a secondary defense, primarily functioning by scavenging free radicals [134]. Additionally, cells possess multiple mechanisms to repair DNA damage caused by reactive ROS. Currently, the focus of antioxidant therapy is on gene therapy and metabolic interventions. Gene therapy can deliver antioxidant genes, modulate pro-inflammatory genes, or introduce insulin secretion-related genes via viral vectors [135]. Metabolic interventions can regulate the intracellular redox state, for example, by using NAD⁺-related compounds. Moreover, interventions that target mitochondrial function or modulate NRF2 to scavenge ROS can also alleviate oxidative stress. NRF2, as a key regulator of antioxidant defense, activates multiple antioxidant enzymes (such as HO-1, NQO1, SOD, and GPX) involved in the regulation of the antioxidant process [136]. It is crucial for combating oxidative stress and serves as a target for most antioxidant drugs.

Mitochondria are one of the primary cellular targets in DN, and mitochondrial dysfunction is widely recognized as a significant factor in the development of DN. Numerous studies have shown that timely and effective amelioration of OS-induced mitochondrial damage is an effective strategy for the treatment of DN. Here, we propose that inhibition of OS by activation of NRF2 improves mitochondrial function, increases the NAD^+^/NADH ratio, and further activates the downstream protein SIRT3 to maintain mitochondrial energy metabolism to reverse macrophage phenotypic changes, thereby reducing inflammation and tissue damage caused by OS during DN.

## Figures and Tables

**Table 1:** Advances in antioxidant research in diabetes mellitus.

Antioxidants	Model	Antioxidant Mechanism	Relative Pathway	References
TP	HFD combined with STZ to establish DN mice, HG-induced MPC5 podocyte	Activation of NRF2	NRF2/HO-1, NRF2/ROS/NLRP3	[56]
SIM	STZ-induced DM rats	Activation of NRF2 and FXR.	NRF2/HO-1, FXR	[57]
Resveratrol	STZ-nicotinamide-induced DM rats, HG-induced mouse podocytes and renal tubular epithelial cells, HG-induced CRL-2573	Maintenance of antioxidant enzyme levels; free-radical scavenging.	Keap1/NRF2, SIRT1-PGC-1α, JNK/NF-κB/NOX/ROS	[58,59,60,61]
Curcumin	STZ-induced rat, HG-induced podocytes	Activation of NRF2; scavenging free radicals; upregulation of enzymatic and non-enzymatic antioxidants.	NRF2, NF-κB, NOX, SOD, PKCβII/p66Shc	[62,63]
α-LA	GK diabetic rats	Mediate the activity of GSH and SOD.	P38/MAPK	[64]
CoQ10	db/db mice, HG-treated mGEC	Antioxidant in mitochondria and lipid membranes.	NRF2/ARE	[65]
EGCG	HFD combined with STZ-induced DN rats, HG-treated NRK-52E	Enhancing NRF2 function and nuclear translocation.	NRF2/Keap1	[66]
Icariin	STZ rats, HG-treated hGMC	Activation of NRF2; increased SOD activity.	NRF2/GPER	[67]
MitoQ	db/db mice	Mitochondria-targeted antioxidants; activation of NRF2.	NRF2/PINK1	[68]
Anthocyanins	db/db mice, HG-treated HK-2 cells	Free-radical scavenging action.	P38 MAPK, ERK1/2	[69]

Abbreviations: TP, triptolide; SIM, simvastatin; α-LA, α-lipoic acid; CoQ10, Coenzyme Q10; EGCG, Epigallocatechin-3-gallate; HG, high-glucose; HFD, high-fat diet; STZ, streptozotocin; GK, Goto–Kakisaki; FXR, farnesoid X receptor; GSH, glutathione; SOD, superoxide dismutase; JNK, c-Jun N-terminal kinase; NRF2, nuclear factor erythroid 2-related factor 2; HO-1, heme oxygenase-1; NLRP3, NOD-like receptor family pyrin domain containing 3; ARE, antioxidant response element; SIRT1, Sirtuin 1; PGC-1α, Peroxisome proliferator-activated receptor gamma coactivator 1-alpha; NF-κB, nuclear factor kappa-light-chain-enhancer of activated B cells; NOX, NADPH oxidase; ROS, reactive oxygen species; P38, p38 mitogen-activated protein kinase; P62, p62 sequestosome 1; Keap1, Kelch-like ECH-associated protein 1; PKCβII, Protein Kinase C beta II; p66Shc, p66 Src homology 2 domain-containing transforming protein; PINK1, PTEN-induced putative kinase 1; MAPK, mitogen-activated protein kinase; ERK1/2, extracellular regulated protein kinases 1/2.
